# Clinical and neuroimaging characterization of the first frontotemporal dementia family carrying the MAPT p.K298E mutation

**DOI:** 10.1007/s10048-024-00756-w

**Published:** 2024-04-09

**Authors:** Federico Emanuele Pozzi, Vittoria Aprea, Ginevra Giovannelli, Francesca Lattuada, Cinzia Crivellaro, Francesca Bertola, Veronica Castelnovo, Elisa Canu, Massimo Filippi, Ildebrando Appollonio, Carlo Ferrarese, Federica Agosta, Lucio Tremolizzo

**Affiliations:** 1 Neurology Department, Fondazione IRCCS San Gerardo Dei Tintori, Monza, Italy; 2 Milan Center for Neuroscience (NeuroMI), University of Milano-Bicocca, Milan, Italy; 3 School of Medicine and Surgery, University of Milano-Bicocca, Milan, Italy; 4 Nuclear Medicine Department, Fondazione IRCCS San Gerardo Dei Tintori, Monza, Italy; 5 Medical Genetics, Fondazione IRCCS San Gerardo Dei Tintori, Monza, Italy; 6 Neuroimaging Research Unit, Division of Neuroscience, IRCCS San Raffaele Scientific Institute, Milan, Italy; 7 Vita-Salute San Raffaele University, Milan, Italy; 8 Neurology Unit, IRCCS San Raffaele Scientific Institute, Milan, Italy

**Keywords:** MAPT, K298E, FTDP-17, Case series, Neuroimaging

## Abstract

We present an in-depth clinical and neuroimaging analysis of a family carrying the MAPT K298E mutation associated with frontotemporal dementia (FTD). Initial identification of this mutation in a single clinical case led to a comprehensive investigation involving four affected siblings allowing to elucidate the mutation's phenotypic expression.

A 60-year-old male presented with significant behavioral changes and progressed rapidly, exhibiting speech difficulties and cognitive decline. Neuroimaging via FDG-PET revealed asymmetrical frontotemporal hypometabolism. Three siblings subsequently showed varied but consistent clinical manifestations, including abnormal behavior, speech impairments, memory deficits, and motor symptoms correlating with asymmetric frontotemporal atrophy observed in MRI scans.

Based on the genotype–phenotype correlation, we propose that the p.K298E mutation results in early-onset behavioral variant FTD, accompanied by a various constellation of speech and motor impairment.

This detailed characterization expands the understanding of the p.K298E mutation's clinical and neuroimaging features, underlining its role in the pathogenesis of FTD. Further research is crucial to comprehensively delineate the clinical and epidemiological implications of the MAPT p.K298E mutation.

## Introduction

Up to 15–20% of hereditary forms of frontotemporal dementia (FTD) are due to microtubule-associated protein tau (*MAPT*) mutations, resulting in hyperphosphorylated neuronal and glial inclusions [1]. More than 50 mutations in the *MAPT* gene are currently known to be the causative factor in a proportion of patients affected by a condition formerly known as frontotemporal dementia and parkinsonism linked to chromosome 17 (FTDP-17) [2]. In this context, the role of the *MAPT* missense mutation p.K298E has been up to now described only in a single clinical case report of a 67-year-old woman, who died at the age of 68, with rapidly progressive non-fluent aphasia associated with gait difficulties [3]. *MAPT* is a neuronally expressed gene consisting of 16 exons subject to differential splicing, leading to six different isoforms depending on the splicing of exons 2, 3, and 10. As exons 9 to 12 constitute the microtubule-binding repeat domain, the three isoforms that include exon 10 are called 4R (4-repeats), while the ones that do not include it are called 3R. Isoforms with 4 repeats show an increased affinity for microtubules, leading to their stabilization, and increase after birth, possibly relating to neuronal maturation and a reduction in neural plasticity [4, 5]. Inadults, 3R and 4R are present in a 1:1 ratio [4], while in primary tauopathies, several synonymous and intronic mutations result in increased inclusion of exon 10, leading to autosomal FTD [6]. The *MAPT* p.K298E (NM_005910.5: c.892A > G; NP_005901.2: p.(Lys298Glu)) occurs in exon 10, right before the beginning of the stem-loop between exon 10 and intron 10 [1].It leads to a change from Lysine to Glutamic Acid at codon 298 and involves a region that has been shown to contain an exonic splice suppressor that down-regulates exon 10 splicing. This results in an increased 4R tau isoform expression by a dominant effect on exon 10 splicing, with a reduced ability to promote microtubule assembly [3]. This is analogous to other missense mutations in the same region, such as p.N296H [3, 4, 7, 8]. It is not clear whether the p.K298E mutation leads to increased tau aggregation, although most mutations in the same region do so, with preferential aggregation of 4R isoforms [1].

**Fig. 1:**
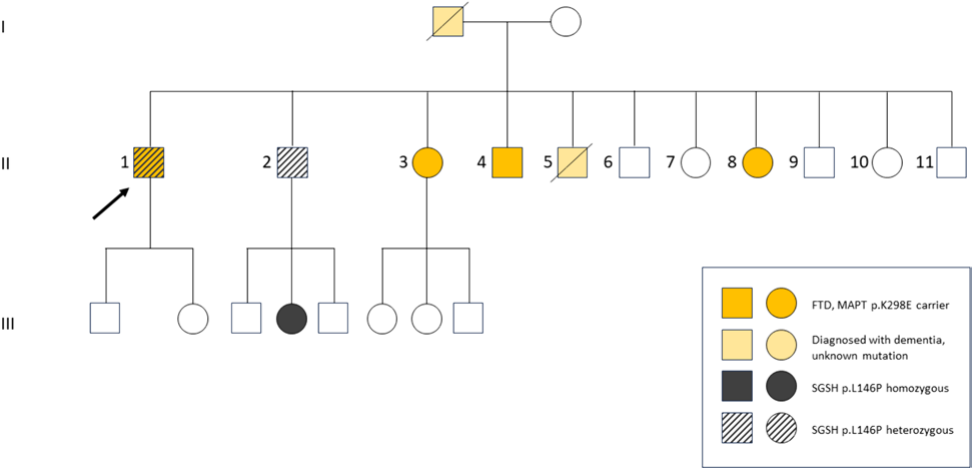
Genetic pedigree of the described family. The proband was case II-1. Other cases described in the text were subject II-2 (also carrying SGSH p.L146P mutation in heterozygosis), II-3, II-4, II-8 and subject II-5 (for whom mutation was not tested). Subject III-4 had Sanfilippo disease

In the first seminal paper, p.K298E mutation pathogenicity was confirmed at the neuropathological examination, showing frontal and anterior temporal lobe atrophy, as well as the caudate nucleus and thalamus involvement, without a side prevalence being reported [3]. Subsequent immunohistochemistry revealed abundant neuronal and glial deposition of hyper-phosphorylated tau with high immunoreactivity for the 4R isoform, while reactivity for 3R tau was much more restricted. Induced neurons obtained from the patient’s skin fibroblast taken 2 h post-mortem showed both 3R and 4R tau isoforms expression [3]. However, besides the elegant work demonstrating a clear-cut pathogenic role for the p.K298E mutation, little clinical information was available for the reported patient and a deeper characterization of the clinical and radiological correlates of this mutation is still needed. We describe here an Italian family carrying the *MAPT* p.K298E mutation and report both clinical, structural and functional neuroimaging in four affected siblings. Figure 1 depicts the family tree, while cases are summarized in Table 1.

**Table 1:** Clinical and radiological features of described p.K298E MAPT mutation cases. The first case is the one presented by Iovino [3]

Case	Sex	Onset	Initial symptoms	Language symptoms	Motor symptoms	Clinical diagnosis	Imaging
[3]	F	65	Progressive gait difficulties, non-fluent aphasia, recurrent falls	Non-fluent aphasia	Bilateral symmetrical myoclonic tremor, increased tone, brisk deep tendon reflexes, progressive gait difficulties	nfvPPA	Cortical atrophy (CT), normal DaT Scan
II-1	M	57	Progressive social isolation and loss of empathy	Mildly impaired sentence comprehension, severe anomia, reduced fluency	Multifocal fasciculations, bradykinesia	bvFTD	Bilateral supratentorial atrophy (CT), frontotemporal hypometabolism with right-side predominance (FDG-PET)
II-4	M	57	Impulsivity, disinhibition, sweet tooth, speech difficulties, short-term memory impairment, depression, poor insight	Semantic and phonemic paraphasia, reduced phonemic fluency	Right limbs weakness, gait instability	bvFTD	Predominantly left frontotemporal and hippocampal atrophy (MRI), left frontotemporal hypometabolism (FDG-PET)
II-8	F	51	Depression, apathy, memory deficits, poor insight	Occasional anomia	Left leg weakness, diffused paratonic rigidity, dyskinesia	rtvFTD	Severe right temporal pole atrophy, mild frontotemporal atrophy (MRI), predominantly right-side fronto-parietal-temporal hypometabolism (FDG-PET)
II-3	F	62	Morpheic seizures, executive and semantic deficits	Semantic deficits	-	bvFTD	Frontal and temporal atrophy at the left side (MRI), left fronto-temporal hypometabolism (FDG-PET)
II-5	M	53	Apathy, inattention, sweet tooth, weird behavior	?	?	?	?

bvFTD: behavioral variant of fronto-temporal dementia. nfvPPA: non-fluent variant of primary progressive aphasia. rtvFTD: right temporal variant of fronto-temporal dementia

Variants have been described according Human Genome Variation Society nomenclature recommendations (version 21.0.0; https://hgvs-nomenclature.org/) [9].

## Case series

### Patient II-1

**Fig. 2:**
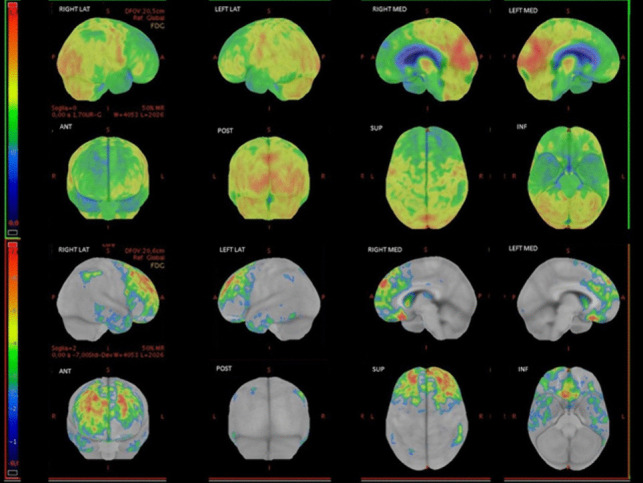
Brain FDG-PET of case II-1, showing predominantly right fronto-temporal hypometabolism. Images have been obtained with CortexID suite (GE Healthcare). Hypometabolic areas appear in cold tones in the upper rows, representing FDG uptake. The lower rows represent the z-score map obtained by comparison with the software database of normal subjects: the higher the z-score (red), the more pronounced the reduction of the metabolism

The proband was a white man referred to our center at the age of 60 because of significant behavioral disorders over the past 3 years (age at onset 57 y.o.). His past medical history was unremarkable, but his father had been diagnosed with dementia, not otherwise specified, at the age of 67. The patient was brought to medical attention because of a progressive loss of interest in social relationships with a decreased level of interaction with his family members and loss of empathy. His wife reported that he had started to be tidier than usual, and she described him as reclusive and silent. He was not agitated or aggressive. He also developed progressive speech difficulties with frequent anomia in the previous years. Upon the first neurological examination, the patient exhibited mild difficulty with sentence comprehension and severe difficulty with naming; he had impaired object knowledge and semantic and phonemic fluencies were both reduced. Repetition of words and sentences, and episodic memory were unaffected. The rest of the neurological examination was unremarkable. Brain MRI was not performed because of claustrophobia, whereas a brain CT scan showed ventricular dilatation and supratentorial atrophy. The patient underwent brain FDG PET which demonstrated frontotemporal hypometabolism, with right-side predominance (Fig. 2). A lumbar puncture was not performed due to the advanced stage of dementia.

Even if there was no clinical evidence for motor or neuromuscular dysfunction, a subsequent muscle ultrasound study was performed and showed occasional multifocal fasciculations in the tibialis anterior muscle, bilaterally, and a single fasciculation in the right biceps brachii and first dorsal interosseous muscles bilaterally.

Given the young age and the family history, genetic testing was proposed. We performed a panel including *C9ORF72*, *GRN*, *FUS*, *TARDBP,* and *PSEN1*, which were all negative; by contrast, *MAPT* direct sequencing revealed the p.K289E mutation in exon 10. One of his younger brothers (II-2) had been identified as an asymptomatic heterozygous carrier of p.Leu146Pro (NM_000199.4: c.437 T > C in exon 4; NP_000190.1: p.(Leu146Pro)) SGSH mutation and his niece had been diagnosed with mucopolysaccharidosis type IIIA (Sanfilippo disease, see Fig. 1). Analogously to his younger brother (II-2), the patient was identified as a heterozygous carrier of p.L146P *SGSH* mutation.

Over the next five years, the patient developed global bradykinesia and became mute and unresponsive to all environmental stimuli.

### Patient II-4

The proband’s brother came to our center at the age of 57 years old; his past medical history was not relevant. At onset, six months before the first visit, the patient started displaying impulsive and disinhibited behaviors. His relatives noticed an increasing and abnormal preference for sweet foods and speech difficulties with occasional semantic and phonemic paraphasia. Moreover, he seemed to be depressed after another brother died of prostate cancer. He also had mild short-term memory impairment with fixation deficits. Upon neurologic examination, the patient had poor insight and minimized his cognitive difficulties. He could not perform the Luria hand sequence even with the examiner; the Go/No Go task showed poor inhibitory control (patient taped like the examiner) and the similarities test revealed impaired abstracting ability. He was also dependent on environmental cues and sensitive to interference; phonemic fluency was reduced (the patient named less than three words starting with the letter F over one minute).

Imaging was done at another institution. Brain MRI showed left predominant frontotemporal and hippocampal atrophy, while FDG-PET confirmed left frontotemporal hypometabolism. The patient refused lumbar puncture. Genetic testing revealed the *MAPT* p.K298E mutation, whereas was negative for the *SGSH* mutation. During the last examination, the patient showed right limbs weakness with gait instability and recurrent falls; he became mute and was treated with Quetiapine and Topiramate to control agitation and hyperphagia.

### Patient II-8

The proband’s sister was seen at our neurological clinic at the age of 52. She had a history of depression and apathy over the past year, with lack of motivation and interest, worsening after her brother died of prostate cancer. Her family members had noticed early memory deficits in daily life (forgetting to turn off the stove or to turn off the tap). Her speech was fluent but with occasional anomia and she exhibited poor awareness about her difficulties.

**Fig. 3:**
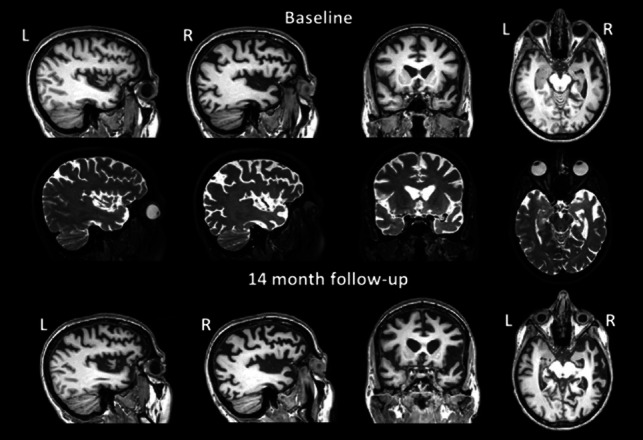
Sagittal, coronal, and axial views of the 3DT1-weighted (first row and third row) and the 3DT2-weighted (second row) image sequences of case II-8 at the first MRI scan and at 14 months of follow-up

**Fig. 4:**
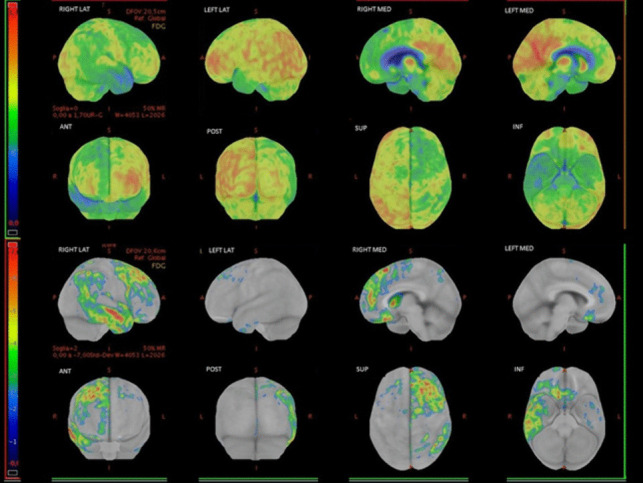
Brain FDG-PET of case II-8, showing right fronto-temporal involvement. Images have been obtained with CortexID suite (GE Healthcare). Hypometabolic areas appear in cold tones in the upper rows, representing FDG uptake. The lower rows represent the z-score map obtained by comparison with the software database of normal subjects: the higher the z-score (red), the more pronounced the reduction of the metabolism

Brain MRI demonstrated severe right temporal pole atrophy with mild right frontotemporal atrophy (Fig. 3). Right temporal atrophy progressed even further on follow-up scans. FDG-PET showed predominantly right-side fronto-parietal-temporal hypometabolism, more evident in the temporal lobe (Fig. 4). The clinical-radiological phenotype was most likely attributable to a right temporal variant of FTD (rtvFTD) [10].

Six months after the first examination, the patient was admitted to our outpatient clinic for progressive left leg weakness. The neurologic examination showed diffused paratonic rigidity, mild weakness of the left lower limb, and distal dyskinesia involving mostly lower limbs, with left-side predominance. She underwent muscle ultrasound without evidence of fasciculations; motor conduction studies were normal and needle EMG showed neither abnormal spontaneous activity nor acute denervation. At 14 months from the first MRI scan, she underwent a further MRI that showed a severe and fast progression of gray matter volume reduction with consequent ventricular enlargement and further atrophy of the right temporal horn, right temporal pole, right Sylvian fissure and perisylvian region (Fig. 3). Genetic analysis confirmed the *MAPT* p.K298E mutation.

### Patient II-3

**Fig. 5:**
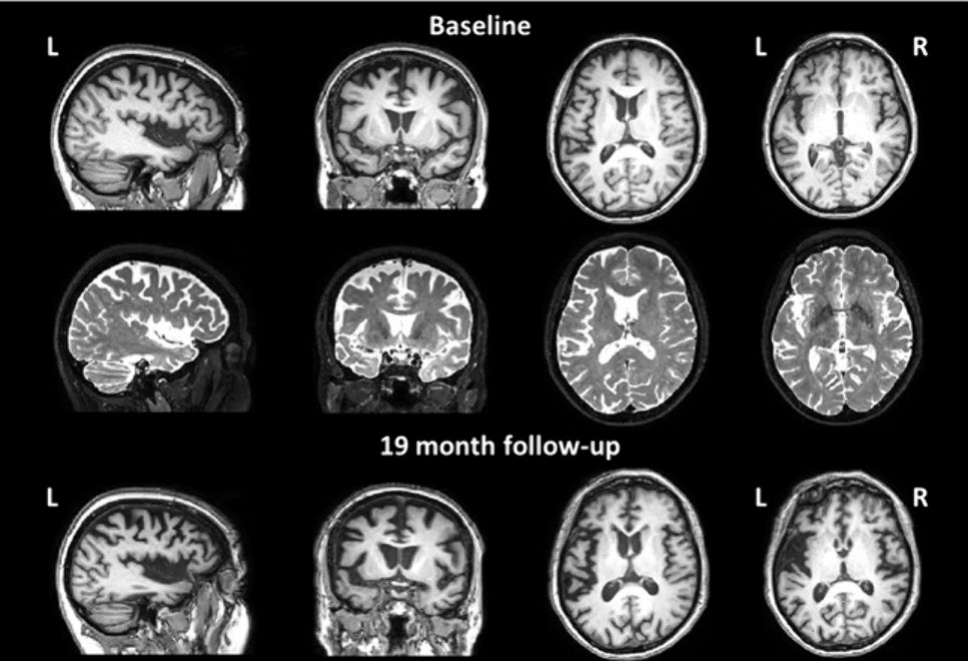
Sagittal, coronal, and axial views of the 3DT1-weighted (first row and third row) and the 3DT2-weighted (second row) image sequences of case II-3 at the first MRI scan and at 19 months of follow-up

**Fig. 6:**
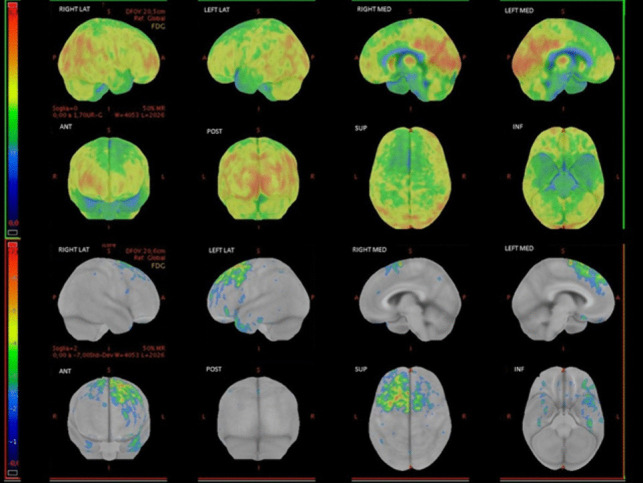
FDG-PET of subject II-3, showing left fronto-temporal hypometabolism. Images have been obtained with CortexID suite (GE Healthcare). Hypometabolic areas appear in cold tones in the upper rows, representing FDG uptake. The lower rows represent the z-score map obtained by comparison with the software database of normal subjects: the higher the z-score (red), the more pronounced the reduction of the metabolism

Two years later, another sister came to our attention at age 62, for two different episodes of morpheic generalized seizures treated with Levetiracetam. A brain MRI scan showed mild frontal and temporal atrophy at the left side, involving the left Sylvian fissure (Fig. 5). FDG-PET revealed left fronto-temporal hypometabolism (Fig. 6).

In the following months, more seizures were documented and progressive cognitive impairment was noted, fulfilling clinical and psychometric (executive and semantic deficits) criteria for probable behavioral fronto-temporal dementia (bvFTD) [11]. At 19 months from the first MRI scan, she underwent a further MRI that showed a moderate pattern of atrophy involving the superior temporal cortex and the temporal pole bilaterally, left Sylvian fissure and left perisylvian brain regions, as well as moderate anterior frontal atrophy (Fig. 5). As expected, genetic analysis confirmed the *MAPT* p.K298E mutation.

### Subject II-5

One proband’s younger brother died at the age of 56 of prostate cancer. He never visited our clinic but from information retrospectively gathered by his relatives, a clinical picture highly suggestive of FTD emerged. At the age of 53, he became progressively apathetic and inattentive, and he developed a craving for sweet foods. No memory loss was reported. His colleagues and relatives noticed “weird behavior” such as cutting meat with two forks or eating without cutlery, or forgetting to wear clothing. The patient was hospitalized in a different center and it was not possible to collect further data.

## Discussion

To the best of our knowledge, this is the first family to be described and carrying the *MAPT* p.K298E mutation. Notably, we describe here the variable clinical presentation and progression with different degrees of severity and neuroimaging appearance, expanding the genotype–phenotype correlations of this mutation. We hypothesize that the *MAPT* p.K298E is associated with early onset (the average onset age was around 57 years old considering all described cases) and a behavioral phenotype (bvFTD), consistent with the predominant phenotype and demographic features of other *MAPT* mutations [8, 12]. Apathy was a common initial behavioral manifestation, predating cognitive impairment. This is in line with a recent study in genetic FTD, showing that apathy is present even in presymptomatic *MAPT* mutation carriers, and is followed by cognitive impairment (but not vice versa) [13].

Interestingly, an initially less prominent speech impairment was present in almost all our patients (anomia, semantic, and phonemic paraphasia), while the case described by Iovino was diagnosed with progressive non-fluent aphasia [3]. Semantic dementia, albeit with behavioral features, is also a possible phenotype of *MAPT* mutations [8]. It should be noticed that an isolated presentation of semantic dementia in MAPT mutations is quite rare, and limited to sporadic case reports [14], and most commonly semantic deficits co-occur with a behavioral phenotype due to a common tropism of MAPT mutations. Analogously, non-fluent aphasia phenotypes have been described with exon 10 *MAPT* mutations [15]. In a GENFI study, 80% of *MAPT*-related FTD exhibited language impairment, with various patterns [16], and naming performances were the worst among genetic FTD forms. Correlates of anomia in *MAPT* mutation carriers were found in the anterior temporal lobes and anterior insula [17]. The described phenotype is probably not unique to MAPT p.K298E mutation, but rather typical of MAPT mutations resulting in a preferential increase of tau 4R isoforms. Indeed, it seems that MAPT mutations resulting in predominant tau 4R isoforms present with linguistic features more often than mutations leading to predominant 3R isoforms, as shown by a recent systematic review [18]. This is also supported by neuropathological data showing a higher burden of tau lesions in case of predominant 4R isoforms compared to predominant 3R isoforms, with distinct tropism for different cerebral regions [19].

Clinical follow-up revealed motor symptoms development in all patients, usually contralateral to the most affected side at neuroimaging. Patients presented with bradykinesia, limb weakness, gait instability, and, in one case, distal dyskinesia; muscle ultrasound performed in our center did not show significant fasciculations. Elements of parkinsonism and basal ganglia degeneration were noted also in the case of *MAPT* p.K298E mutation described by Iovino in 2014 (notably, with a normal DaT-Scan). While extensive phosphorylated tau was reported, also affecting the caudate nucleus and the substantia nigra (which seems typical for mutations in exon 10 of *MAPT* [8, 20]), no α-synuclein pathology was present in her brain [3]. Moreover, extrapyramidal symptoms have been described in the context of *MAPT* mutations affecting exon 10 splicing [8], and an abnormal striatal tau isoform content with an excess of 4R isoforms seems responsible for clinical parkinsonism in murine models of tauopathy resulting from altered exon 10 splicing [21]. Therefore, the parkinsonian features also present in our cases might arise from a tau-related degeneration of key extrapyramidal regions.

Motor symptoms in our family were not limited to extrapyramidal features, but upper and lower motor signs were not sufficient to pose a diagnosis of amyotrophic lateral sclerosis (ALS) in any case [22, 23]. Indeed, a constellation of nonspecific motor signs like bradykinesia and ALS-like features such as spasticity, limb weakness, and fasciculations are present in around 40% of *MAPT* mutation carriers, although less common than the Parkinson’s-like motor phenotype [24].

In two cases, mild memory impairment was noticed. This is also consistent with previous literature showing impaired performance on memory tasks even in presymptomatic *MAPT* mutation carriers [25].

Brain MRI showed asymmetrical frontotemporal atrophy and the metabolic PET confirmed asymmetrical frontotemporal involvement, with greater right involvement in two patients and left-side predominance in the other two patients. Indeed, *MAPT* mutations affecting exon 10 splicing seem to be predominantly associated with structural and metabolic medial temporal lobe involvement [8]. In the aforementioned GENFI cohort, the temporal pole was atrophic in up to 70% of *MAPT* mutation carriers, significantly differing from other mutation groups [16]. Temporal lobe volumes show the fastest decline over time even in presymptomatic *MAPT* mutation carriers [26], and it was even more accelerated in those who converted to dementia, who also showed frontal and parietal longitudinal atrophy [27]. Mesial temporal gray matter atrophy may be present in presymptomatic carriers as early as their thirties [28]. However, the pattern of atrophy seems to be dependent on the specific *MAPT* mutation, with distinct clinic-radiological peculiarities [29].

It would be interesting to speculate on the role of SGSH heterozygosity as a potential risk factor for neurodegeneration, as is the case for mutations in other genes involved in lysosome storage disorder, such as those in glucosaminidase (*GBA*) and Parkinson’s disease. *SGSH* mutations are responsible for mucopolysaccharidosis type IIIA, the most common form of Sanfilippo syndrome, an autosomal recessive disorder of lysosome storage with onset in childhood [30]. It results in developmental delay arising between 2 and 6 years of age, with subsequent cognitive decline, sleep disorders, hyperactivity, and aggressive behavior, and finally, loss of mobility, swallowing troubles, and epilepsy, leading to premature death [31, 32]. The p.L146P is a missense mutation previously described only in one Italian patient, drastically changing the protein structure, and leading to a severe phenotype [33]. Neurodegeneration in mucopolysaccharidosis III has been demonstrated independently of the specific genetic mutation [34–36], being the result of axonal dystrophy with an accumulation of ubiquitin-positive lesions containing phosphorylated-tau, wildtype α-synuclein, APP and β-amyloid, as shown by clinical and preclinical evidence [37–39]. However, mice carrying heterozygous p.D31N mutation in *SGSH* did not show an increased presence of brain pathology or neurodegeneration compared to controls, nor a significant accumulation of heparan sulfate despite a 50% reduction in SGSH levels. Nevertheless, they exhibited significantly worse performances on the negative geotaxis test and significant increase in the length of the outer-most dendritic processes with aging [40]. While this would support the idea that p.D31N *SGSH* heterozygosity does not overtly hasten age-related neurological decline, it is possible that such a decline, if indeed present, might be mutation-specific. Regrettably, we do not have enough data to evaluate whether heterozygous carriers of p.L146P mutation exhibit worse neurological outcomes. Hence, despite the speculations, the most plausible explanation for the simultaneous presence of the SGSH p.L146P heterozygous mutation and MAPT p.K298E mutations in the proband is likely mere coincidence.

Our report has some limitations. Pathological confirmation was not available, although almost all the cases satisfy the criteria for bvFTD [11], with the exception of case II-8, who was most likely affected by rtvFTD. Analogously, we could not explore brain tau accumulation in vivo with tau-PET, which shows preclinical alterations in other *MAPT* mutations [41], as this was not available at our institution. Moreover, given either the advanced stage of dementia at presentation or the refusal of subjects involved, it was not possible to explore CSF or blood biomarkers in this family.

In conclusion, the p.K298E mutation belongs to a small group of exon 10 mutations and it is responsible for early onset bvFTD with a variegated phenotype, including language and motor symptoms, with predominant fronto-temporal hypometabolism. At present, it is necessary to implement our knowledge about *MAPT* p.K298E to better clarify its clinical and epidemiological role. Continuous follow-up of the family described in this paper, and the identification of other families, will help increase our understanding of *MAPT* exon 10 mutations.

## Data Availability

The original contributions presented in the study are included in the article, further inquiries can be directed to the corresponding author.
